# Mollugin Has an Anti-Cancer Therapeutic Effect by Inhibiting TNF-α-Induced NF-κB Activation

**DOI:** 10.3390/ijms18081619

**Published:** 2017-07-26

**Authors:** Zhe Wang, Ming Yue Li, Chunliu Mi, Ke Si Wang, Juan Ma, Xuejun Jin

**Affiliations:** Key Laboratory of Natural Resources of Changbai Mountain & Functional Molecules, Ministry of Education, Molecular Medicine Research Center, College of Pharmacy, Yanbian University, Yanji 133002, China; megaton0514@gmail.com (Z.W.); 18243390885@163.com (M.Y.L.); mi.chunliu@163.com (C.M.); kesiwangchinese@126.com (K.S.W.)

**Keywords:** mollugin, cancer, NF-κB, NF-κB target genes

## Abstract

The NF-κB signaling pathway plays a pivotal role in regulating the immune response and inflammation. However, it has been shown that NF-κB also has a major role in oncogenesis. Therefore, NF-κB inhibitors have been considered as potential drugs against cancer. Herein, we searched for NF-κB inhibitors from natural sources and identified mollugin from the roots of *Rubia cordifolia* L. as an inhibitor of NF-κB activation. We found that mollugin significantly inhibited the expression of an NF-κB reporter gene induced by tumor necrosis factor (TNF)-α in a dose-dependent manner. Moreover, mollugin inhibited TNF-α-induced phosphorylation and nuclear translocation of p65, phosphorylation and degradation of inhibitor of κB (IκBα), and IκB kinase (IKK) phosphorylation. Furthermore, we discovered that pretreatment of cells with mollugin prevented the TNF-α-induced expression of NF-κB target genes, such as genes related to proliferation (COX-2, Cyclin D1 and c-Myc), anti-apoptosis (Bcl-2, cIAP-1 and survivin), invasion (MMP-9 and ICAM-1), and angiogenesis (VEGF). We also demonstrated that mollugin potentiated TNF-α-induced apoptosis and inhibited proliferation of HeLa cells. We further demonstrated in vivo that mollugin suppressed the growth of tumor xenografts derived from HeLa cells. Taken together, mollugin may be a valuable candidate for cancer treatment by targeting NF-κB.

## 1. Introduction

Cancer is one of the most common causes of mortality and morbidity today, with approximately 14 million new cases in 2012 and about 8.8 million deaths in 2015. Chemotherapy is one of the important approaches among cancer therapies. However, treatments using specific agents or inhibitors that target only one biological event or a single pathway usually fail in cancer therapy. Thus, combination treatments attacking or blocking multiple important pathways have been considered to overcome this failure [1]. Searching for novel agent targeting pathways that are responsible for promoting cancer cell survival and growth is urgently needed for cancer therapeutic strategies. It has been demonstrated that many natural products and derivatives are potential candidates for cancer therapy, particularly from medicinal plants used in traditional Chinese medicine [2]. Therefore, a search for new therapeutic agents from natural products is necessary for the treatment of human cancer.

Mollugin, a type of naphthohydroquinone, is a major bioactive component isolated from *Rubia cordifolia* L., which has been used as a traditional Chinese medicine for centuries and is officially listed in the Chinese Pharmacopoeia. It has been used for the treatment of coughs, joint inflammation, uterine hemorrhage, and uteritis [3]. Previous investigations have shown that mollugin has various biological activities in the anti-inflammatory and anti-tumor fields [4,5,6]. However, the molecular mechanism behind the anti-tumor effects of mollugin has not been fully elucidated.

The transcription factor, NF-κB, is a major regulator of the immune response, and is associated with the development and progression of diseases such as autoimmune diseases and cancer [7]. The NF-κB family consists of five members: RelA (p65), RelB, c-Rel, p50/p105 (NF-κB1), and p52/p100 (NF-κB2). These members can form complexes either as homodimers or heterodimers. The prototypical complex mostly referred to as “NF-κB” is the p65/p50 dimer. In most cell types, NF-κB complexes are retained in the cytoplasm by a family of inhibitory proteins known as inhibitors of NF-κB (IκBs) [8]. In response to a variety of stimuli, such as the binding of tumor necrosis factor (TNF)-α to its membrane receptor, IκBα is phosphorylated at Ser32/Ser36 by IκB kinase (IKK). IKK is a multi-subunit kinase complex, typically composed of IKKα and IKKβ, and two molecules of IKKγ/NF-κB essential modulator (NEMO) [9]. Phosphorylated IκB is then degraded by the proteasome, which allows NF-κB dimers to translocate to the nucleus, where they stimulate the expression of target genes.

It has been reported that NF-κB promotes the migration and metastasis of several kinds of cancer cells, and that it plays a central role in the regulation of many genes, including those involved in immunity and inflammation, anti-apoptosis, cell proliferation, and tumorigenesis [10,11]. NF-κB regulates numerous genes associated with proliferation, including cyclooxygenase-2 (COX-2), cyclin D1, and c-Myc, genes associated with anti-apoptosis (such as cellular inhibitor of apoptosis protein 1 (cIAP-1), B-cell lymphoma-2 (Bcl-2) and survivin), and genes required for invasion and angiogenesis such as matrix metalloproteinase (MMP-9), intercellular cell adhesion molecule-1 (ICAM-1), and vascular endothelial growth factor (VEGF) [12,13]. Because NF-κB is usually activated in cancer cells and is usually involved in the survival of cancer cells, blocking NF-κB is expected to reduce the survival threshold. Therefore, NF-κB inhibition is being tested mainly for use with chemo-and radiotherapy [14].

In the present study, we explored the anti-tumor effect of mollugin through the inhibition of the NF-κB pathway. We showed that mollugin inhibited the expression of an NF-κB reporter gene induced by TNF-α in a dose-dependent manner, and that mollugin inhibited TNF-α-induced phosphorylation and nuclear translocation of p65, phosphorylation and the degradation of IκBα, and IKK phosphorylation. We also demonstrated that mollugin potentiated TNF-α-induced apoptosis and inhibited proliferation of HeLa cells. Our findings highlight the anti-tumor potential of mollugin, a traditional Chinese medicine, which may be a novel therapeutic agent against human cancer.

## 2. Materials and Methods

### 2.1. Cell Culture and Reagents

**Figure 1:**
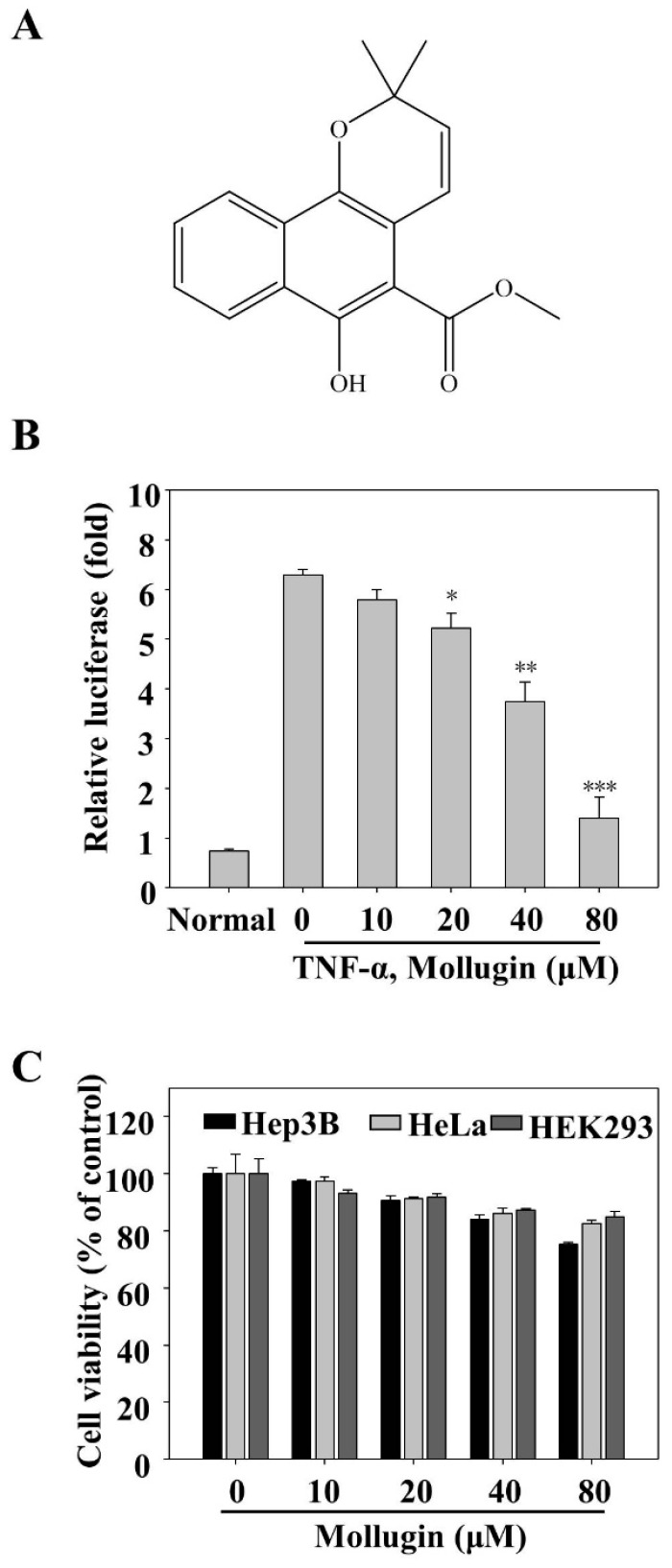
Mollugin inhibits the TNF-α-induced NF-κB-dependent reporter gene expression. (**A**) Molecular structure of mollugin; (**B**) HeLa cells were transiently co-transfected with a pNF-κB-Luc plasmid for 24 h, followed by pretreatment with mollugin for additional 12 h. Then, the cells were stimulated for 12 h with TNF-α (10 ng/mL), and the luciferase activity was determined, as previously mentioned, in “materials and methods”. Data represented as mean ± SD from three replicates, * *p* < 0.05; ** *p* < 0.01; *** *p* < 0.001 compared to the control group; (**C**) HeLa, Hep3B, and HEK293 cells were treated with different concentrations of mollugin, as shown in the figure. After 24 h incubation, cell viability was measured by MTT assay. Representative data from three independent experiments with similar outcomes is shown.

HeLa, Hep3B, and HEK293 cells were acquired from the American Type Culture Collection (Manassas, VA, USA) and maintained in Dulbecco’s modified Eagle’s medium (DMEM) supplemented with 10% fetal bovine serum (FBS; Gibco, Grand Island, NY, USA) and 1% penicillin/streptomycin (Invitrogen, Carlsbad, CA, USA) at 37 °C in a humidified incubator containing 5% CO_2_ atmosphere. TNF-α was obtained from R&D Systems (Minneapolis, MN, USA). Mollugin was purchased from the National Institutes for Food and Drug Control (NIFDC, Beijing, China) with purity of at least 98% in HPLC analysis, and its structure is also shown in Figure 1A. Primary antibodies against IκBα, phospho-IκBα (Ser32), p65, phospho-p65 (Ser536), poly ADP-ribose polymerase (PARP), caspase-3, cIAP-1, IKKα/β, and phospho-IKKα (Ser176)/IKKβ (Ser177) were obtained from Cell Signaling Technology (Beverly, MA, USA). Antibodies against COX-2, cyclinD1, Bcl-2, survivin, MMP-9, ICAM-1, VEGF, and Topo-I were purchased from Santa Cruz Biotechnology (Santa Cruz, CA, USA). The antibody against α-tubulin was purchased from Sigma (St. Louis, MO, USA). Other cell culture reagents were all obtained from commercial companies.

### 2.2. Plasmids, Transfections, and Luciferase Assay

The Dual Luciferase Reporter Assay system was employed to determine the NF-κB-dependent luciferase activity and transfection was performed, as previously described [15]. HeLa cells were seeded in a 96-well plate at a density of 1 × 10^5^ cells/well for 24 h, and then transfected with pNF-κB-Luc plasmid (Strategene, LaJolla, CA, USA). After 24 h incubation, the cell culture medium was replaced with a fresh medium containing various concentrations of mollugin and incubated for 12 h, followed by stimulation with 10 ng/mL of TNF-α for 12 h. Luciferase activity was quantified by Luminoskan^TM^ Ascent Microplate Luminometer (Thermo Scientific, Waltham, MA, USA) using 100 μL of assay buffer containing luciferin. The data from NF-κB-dependent luciferase reporter was normalized by co-transfection with pRL-CMV (Promega, Madison, WI, USA), which expresses Renilla luciferase.

### 2.3. Measurement of Cell Viability by MTT Assay

HeLa, Hep3B, and HEK293 cells were seeded at a density of 1 × 10^5^ cells per well and cultured overnight. After incubation, the cells were treated with various concentrations of mollugin for 24 h. The supernatant was removed and the cells were labeled with 3-(4,5-dimethyl-2-thiazolyl)-2,5-diphenyl-2-*H*-tetrazolium bromide (MTT) (Sigma, St. Louis, MO, USA) solution at a final concentration of 0.75 mg/mL for 4 h. Then, 100 μL of DMSO was added into the 96-well plate to dissolve the formazan. The cell viability was determined by measuring the absorbance at 570 nm using Multiskan GO (Thermo Electron Corp., Marietta, OH, USA).

### 2.4. Apoptosis Assays

Apoptosis assays were operated by the method previously described [16]. Annexin V staining was performed using the Annexin V-FITC Apoptosis Detection Kit (Becton-Dickinson Biosciences, San Diego, CA, USA) according to the manufacturer’s instructions. Briefly, HeLa cells were collected after incubation and washed with phosphate buffered solution (PBS). Afterward, the cells were centrifuged and incubated with Annexin V-FITC and 2 μg/mL propidium iodide in binding buffer containing 10 mM Hepes, pH 7.4, 140 mM NaCl, and 2.5 mM CaCl_2_ for 15 min at 37 °C in the dark. The apoptotic cells were analyzed by flow cytometry using a FACScan flow cytometer. The CellQuest software (Becton-Dickinson Biosciences, San Diego, CA, USA) was used for data analysis.

### 2.5. Western Blot Analysis

The method used for Western Blot analysis has been described previously [17]. HeLa cells were cultured using 6 cm-dishes for 24 h before performing this assay. Then, the cells were incubated with indicated concentrations of mollugin for 12 h in the absence or presence of 10 ng/mL TNF-α stimulation. Cells were disrupted to collect whole-cell extracts in pre-cold lysis buffer (50 mM Tris-HCl, pH 7.5, 1% Nonidet P-40, 1 mM EDTA, 1 mM phenylmethyl sulfonylfluoride) containing the protease inhibitor cocktail (BD Biosciences, San Diego, CA, USA). In certain experiments, the collection of nuclear extracts was achieved by using NE-PER reagent. Determination of protein concentration was performed using the Bradford method. Fifty μg of whole-cell extracts or thirty μg of nuclear extract protein per lane was separated by SDS-polyacrylamide gels (8–15%) and then the protein was transferred to a polyvinylidene difluoride membrane (Millipore, Bedford, MA, USA). After blocking the membrane with 5% skim milk, the protein was probed with the target antibody and visualized by enhanced chemiluminescence following the instructions of the manufacturer (Amersham Pharmacia Biotec, Buckinghamshire, UK).

### 2.6. Immunofluorescence of NF-κB p65

HeLa cells were seeded into 24-well plates at 1 × 10^4^ cells/well for 24 h. Then, cells were treated with mollugin (80 μM) for 12 h (Cells treated with DMSO and 10 ng/mL TNF-α alone were used as negative and positive control, respectively), followed by treatment with 10 ng/mL TNF-α for 30 min. After treatment, cells were rinsed once in PBS, followed by fixation in fresh 4% paraformaldehyde for 30 min at room temperature. Cells were permeabilized with 0.2% Triton X-100 in PBS at room temperature. Next, the cells were incubated with PBS containing 5% BSA for 30 min, and then incubated with the primary antibody against NF-κB p65 at 4 °C overnight. On the second day, the cells were incubated with Alexa flour^®^ 488 goat anti-rabbit lgG (H + L) for 30 min at room temperature, followed by DAPI staining for 30 min before observation. The staining was examined using the Olympus IX83 inverted fluorescence microscope (Olympus Corporation, Tokyo, Japan) at 40× magnification (scale bar 20 μm). The p65 protein showed color in green and the nuclei showed in blue. The merged images were conducted using Image J software (Wayne Rasband National Institutes of Health, Bethesda, MD, USA) to show co-localization (cyan fluorescence).

### 2.7. Reverse Transcription-Polymerase Chain Reaction (RT-PCR)

HeLa cells were cultured in 6-cm culture plates to confluence. Then, the cells were treated with indicated concentrations of mollugin with or without 10 ng/mL TNF-α. Total RNA was collected using RNeasy Mini kits according to the manufacturer’s instructions (Qiagen, Valencia, CA, USA). Then, 1 μg of total RNA was reversely transcribed to complementary DNA using a 20 μL reverse transcription reaction mixture according to the manufacturer’s protocol (TaKaRaBio, Kyoto, Japan). Primer pairs used for RT-PCR amplification were as follows: cyclin D1, 5′-CTGGCCATGAACTACCTGGA-3′, and 5′-GTCACACTT GATCACTCTGG-3′; c-Myc, 5′-CTCTCAACGACAGCAGCCCG-3′, and 5′-CCAGTCTCAGACCTAGTGGA-3′; VEGF, 5′-GCTCTACCTCCACCATGCCAA-3′, and 5′-TGGAAGATGTCCACCAGGGTC-3′; GAPDH, 5′-ACCAGGTGGTCTCCTCT-3′, and 5′-TGCTGTAGCCAAATTCGTTG-3′. GAPDH was used as the housekeeping gene control. PCR products were resolved on 3% agarose gel by electrophoresis and then labelled using ethidium bromide. The stained bands were viewed under UV light.

### 2.8. EdU Labeling and Immunofluorescence

The cells were cultured in 96-well plates, and then cells were treated with 80 μM mollugin. After 12 h incubation, the cells were labelled with 5-ethynyl-2′-deoxyuridine (EdU, RIBOBIO; R11053) for 1 h and stained with Apollo^®^ 567 following the instructions of manufacturer. The data was measured using the Olympus IX83 inverted fluorescence microscope (Olympus Corporation, Tokyo, Japan).

### 2.9. Colony Formation Assay

HeLa cells were seeded in 6-well plates for 24 h. Then, the cells were treated with various concentrations of the mollugin for 12 h. Subsequently, the media was replaced with drug-free media and culture cells for an additional 10 days, until colonies were obviously visible and countable. Then, the colonies were fixed with 4% formaldehyde in PBS and stained with 0.02% crystal violet.

### 2.10. In Vivo Xenograft Assay

The entire performance of surgeries and care were conducted on the animals according to IACUC guidelines. Six-week-old specific-pathogen-free Crj:BALB/c female athymic nude mice (Vital River, Beijing, China) were randomly divided into three groups (*n* = 5 per group). All of the mice were subcutaneously injected with 0.2 mL HeLa cells (5 × 10^7^ cells/mL) for preparation of xenograft tumor models. Then, they were orally administered suspension of Mollugin in saline three times per week, at a dose of 25 and 75 mg/kg body weight. Tumor size was calculated every three days and tumor volume was calculated based on the formula: (length × (width)^2^)/2. Finally, all tumors were taken out from sacrificed mice and solidified for further tests. Experimental protocol was approved by the Committee on the Ethics of Animal Experiments of the Yanbian University (Permit Number: SCXK-JI-2011-0007, 6 November 2011) in accordance with the National Institute of Health Guide for the Care and Use of Laboratory Animals.

### 2.11. Data Analyses

All results are expressed as mean ± SD. Comparisons among groups were performed with one-way ANOVA and Tukey’s multiple comparison tests (Graphpad Software, Inc., San Diego, CA, USA). A *p* value less than 0.05 was considered statistically significant.

## 3. Results

### 3.1. Mollugin Inhibits the TNF-α-Induced Expression of an NF-κB Reporter Gene

To explore potential NF-κB inhibitors from natural products, we investigated mollugin, a major bioactive component of *Rubia cordifolia*, which has been used as a traditional Chinese medicine for centuries. To investigate whether this compound affected the TNF-α-induced NF-κB activation, an NF-κB reporter gene assay was conducted. The cells were transfected with a pNF-κB-Luc plasmid and then treated with TNF-α containing the indicated concentrations of mollugin. As expected, the NF-κB reporter gene expression was significantly inhibited by mollugin, in a dose-dependent manner (Figure 1B). To test the cellular toxicity of mollugin, an MTT assay was performed. Mollugin (up to 80 μM) did not display significant cellular toxicity in HeLa, Hep3B, and HEK293 cells (Figure 1C).

### 3.2. Mollugin Inhibits TNF-α-Induced Phosphorylation and Nuclear Translocation of p65, Phosphorylation and Degradation of IκBα, and IKK Phosphorylation

**Figure 2:**
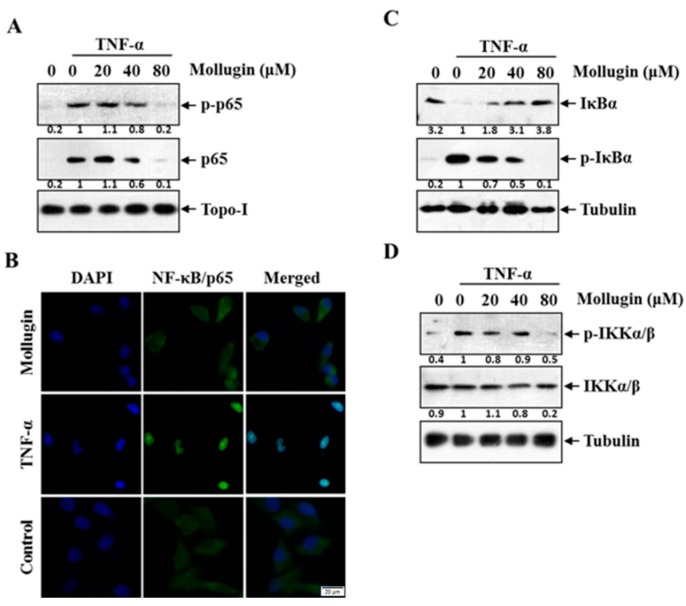
Mollugin inhibits TNF-α-induced phosphorylation and nuclear translocation of p65, phosphorylation and degradation of IκBα, and IKK phosphorylation. (**A**) HeLa cells were stimulated with 10 ng/mL TNF-α for 30 min after pretreatment of mollugin (20 μM, 40 μM, and 80 μM) for 12 h. The extracts of nucleus were analyzed using Western blot with indicated antibodies against p-p65, p65, and Topo-I; (**B**) HeLa cells were stimulated with 10 ng/mL TNF-αstimulation for 30 min after pretreatment of 80 μM mollugin for 12 h. When being fixed by 4% paraformaldehyde, cells were firstly probed by specific anti-p65 antibody and then Alex Flour^®^ 488 (green). The blue counterstained nucleus (with DAPI) was examined by Fluorescence microscopy. Scale bars: 20 μm. All images were obtained for each fluorescence channel by using suitable filters with 40× objective. The merged images were performed using Image J software; (**C**) HeLa cells were stimulated with 10 ng/mL TNF-α for 30 min after pretreatment of mollugin (20 μM, 40 μM, and 80 μM) for 12 h. The extracts of cytoplasm were analyzed by Western blot using indicated antibodies against p-IκBα, IκBα, and tubulin; (**D**) HeLa cells were stimulated with TNF-α (10 ng/mL) for 30 min after pretreatment of mollugin (20 μM, 40 μM, and 80 μM) for 12 h. The extracts of cytoplasm were analyzed by Western blot by using indicated antibodies against p-IKKα/β, IKKα/βand tubulin.

Since the phosphorylation and nuclear translocation of p65 is vital to the NF-κB transcriptional activity [18], the effects of mollugin on TNF-α-induced phosphorylation and nuclear translocation of p65 were examined. HeLa cells were pretreated with the indicated concentrations of mollugin for 12 h, and then stimulated with TNF-α (10 ng/mL) for 30 min. Nuclear extracts were analyzed by western blot and the result is shown in Figure 2A. Mollugin could significantly inhibit the TNF-α-induced p65 phosphorylation and block TNF-α-induced nuclear translocation of p65 in a dose-dependent manner. To confirm these results, immunofluorescence was performed to examine p65 translocation in HeLa cells. The results showed that p65 localized in the cytoplasm of untreated cells and in cells pre-treated with mollugin (80 μM) followed by TNF-α treatment, whereas it translocated to the nucleus in cells treated with TNF-α alone (Figure 2B).

A key step in the NF-κB signaling pathway is IκB degradation, which leads to the release of bound NF-κB for subsequent nuclear translocation. To determine whether the mollugin inhibition of p65 nuclear translocation resulted from phosphorylation and degradation of IκBα, we exposed cells pretreated with or without mollugin to TNF-α for 30 min. IκB-α was obviously degraded upon TNF-α stimulation, and the degradation process was completely inhibited by 80 μM mollugin (Figure 2C).

IκBα phosphorylation requires IKK activation. To examine the effect of mollugin on IKK activation, we investigated whether mollugin influences TNF-α-induced IKK phosphorylation. Treatment with 80 μM mollugin abolished the TNF-α-induced IKK phosphorylation. However, both the TNF-α and mollugin treatments had a weak effect on IKK protein expression (Figure 2D).

### 3.3. Mollugin Inhibits the Expression of TNF-α-Induced NF-κB-Regulated Genes

**Figure 3:**
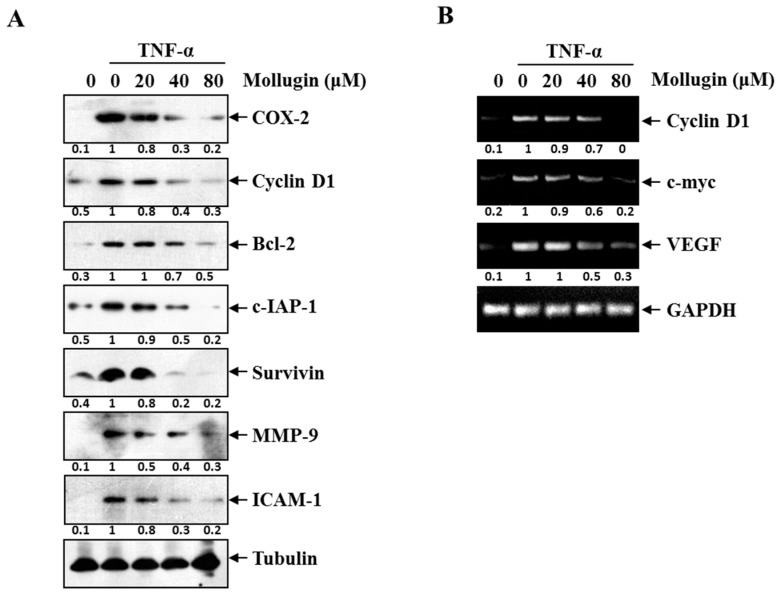
(**A**) HeLa cells were pre-incubated of mollugin (20, 40, and 80 μM) for 12 h prior to stimulation with 10 ng/mL TNF-α for another 12 h. Whole cell extracts were analyzed by Western blot assay for COX-2, Cyclin D1, Bcl-2, cIAP-1, survivin, MMP-9, ICAM-1, and tubulin; (**B**) HeLa cells were pre-incubated of mollugin (20, 40, and 80 μM) for 12 h prior to stimulation with 10 ng/mL TNF-α for another 12 h. Total RNA was analyzed by RT-PCR for Cyclin D1, c-Myc, and VEGF. GAPDH was used as an equal loading control.

COX-2, Cyclin D1, and c-Myc regulate cellular proliferation and are regulated by NF-κB. NF-κB upregulates the expression of numerous genes implicated in facilitating tumor cell survival, such as Bcl-2, c-IAP1, and survivin. NF-κB also regulates the expression of genes involved in invasion (MMP-9 and ICAM-1) and angiogenesis (VEGF). Thus, the effect of mollugin on the expression of these NF-κB-regulated genes and proteins were examined. After HeLa cells were stimulated with 10 ng/mL TNF-α for 12 h in the presence or absence of mollugin, the expression of the proliferation proteins (COX-2 and Cyclin D1), the anti-apoptotic proteins (Bcl-2, c-IAP1, and survivin), and the invasion and angiogenesis proteins (MMP-9 and ICAM-1) were analyzed by western blotting. Mollugin markedly suppressed the TNF-α-induced expression of all these proteins in a dose-dependent manner (Figure 3A). Similarly, 80 μM mollugin inhibited the TNF-α-induced mRNA expression of *Cyclin D1*, *c-Myc*, and *VEGF* (Figure 3B).

### 3.4. Mollugin Potentiates TNF-α-Induced Apoptosis

**Figure 4:**
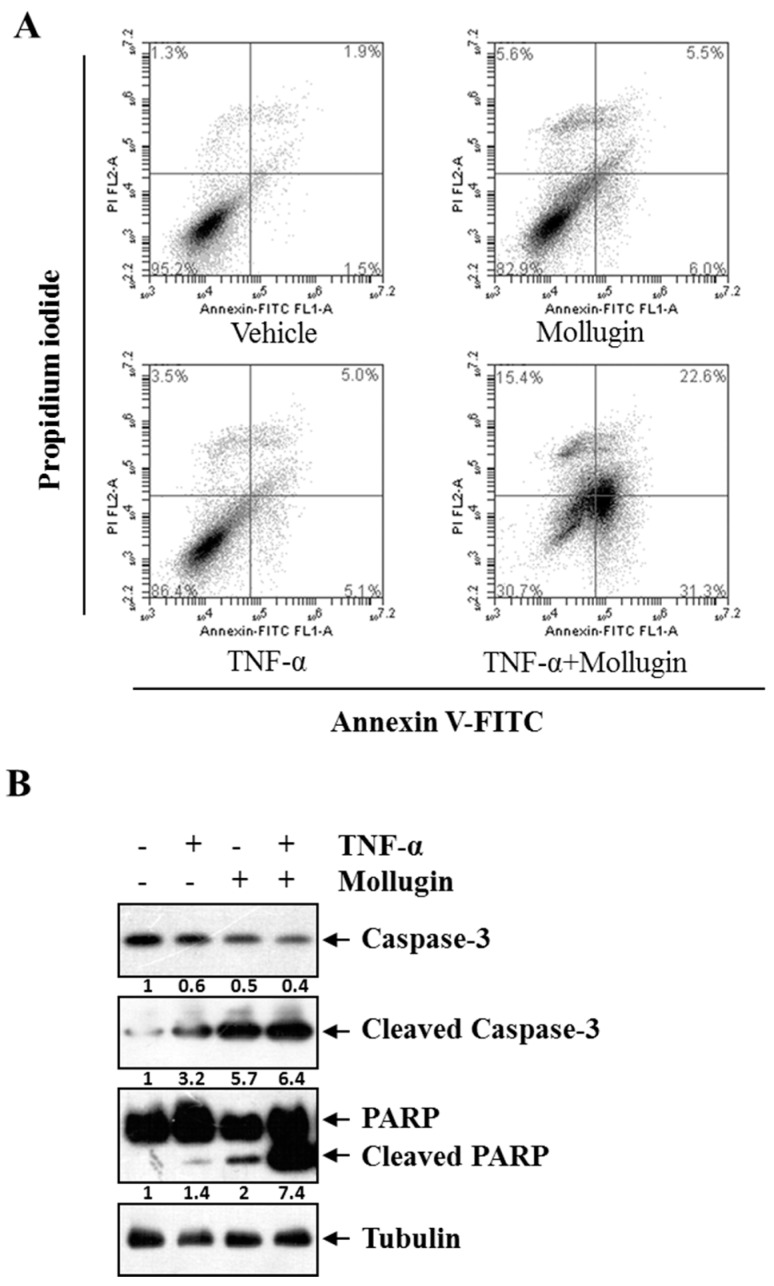
Mollugin potentiates TNF-α-induced apoptosis. (**A**) HeLa cells were pre-incubated in the presence of 80 μM mollugin for 12 h prior to treatment with 10 ng/mL TNF-α for 24 h. Then, the cells were collected and labelled with Annexin V-FITC and propidium iodide for staining. The stained cells were analyzed using a flow cytometer. The lower right quadrant represented the early apoptotic cell (Annexin-V+ and PI−) and the upper right quadrant represented the late apoptotic cells (Annexin-V+ and PI+); (**B**) HeLa cells were pre-incubated in the presence of 80 μM mollugin for 12 h prior to treatment with 10 ng/mL TNF-α for 24 h. Western blot assay was performed for the total and cleaved capase-3, PARP. Tubulin was used as an internal control.

Next, we examined whether mollugin enhances TNF-α-induced apoptosis. Annexin V/propidium iodide (PI) double staining showed that mollugin potentiated TNF-α-induced apoptosis. Figure 4A showed that if HeLa cells were treated only with vehicle, TNF-α, or mollugin, the percentage of apoptosis was at a low level (3.4% (vehicle), 10.1% (TNF-α), and 11.5% (mollugin), respectively). However, a sharp increasing apoptosis (53.9%) could be induced by combined treatment with TNF-α and mollugin. Because caspase is a group of cysteine proteases that are essential for eukaryotic cell apoptosis [19]; we investigated whether mollugin affects TNF-α-induced activation of caspase-3. Treatment with either TNF-α or mollugin slightly affected the caspase-3 activation, whereas co-treatment with both TNF-α and mollugin potentiated caspase-3 activation, as indicated by the presence of cleaved caspase-3 (Figure 4B, top panel). We then determined whether mollugin affects TNF-α-induced poly ADP ribose polymerase (PARP) cleavage. Again, mollugin potentiated the effect of TNF-α-induced PARP cleavage (Figure 4B, middle panel). These results showed that mollugin enhanced the apoptotic effects of TNF-α.

### 3.5. Mollugin Inhibits Cell Proliferation

**Figure 5:**
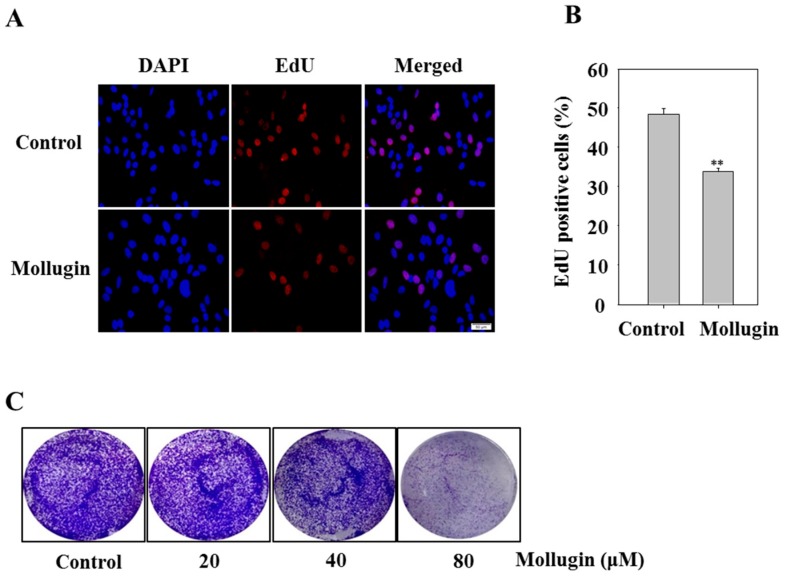
Effect of mollugin on HeLa cell proliferation. (**A**,**B**) Hela cells were treated with 80 μM mollugin for 12 h. After EdU labeling, the data were collected using fluorescent microscope at 20× magnification (scale bar: 50 μm); ** *p* < 0.01 compared to control group; (**C**) HeLa cells were treated with indicated concentrations of mollugin (20, 40, and 80 μM) for 12 h, and then the medium was replaced by a fresh medium. Cells were allowed to grow for 10 days and subjected to crystal violet staining of cell growth. The diameter of each well was 34.8 mm.

To investigate whether mollugin affects cell proliferation, the 5-ethynyl-2′-deoxyuridine (EdU) incorporation assay was performed. In the mollugin-treated group, the number of EdU-positive cells was lower than that in the control group, indicating that mollugin inhibited the proliferation of HeLa cells in vitro (Figure 5A,B). To determine the effect of the long-term anti-proliferative activity of mollugin, a clonogenic assay was performed. The clonogenicity of HeLa cells in the mollugin group decreased in a concentration-dependent manner (Figure 5C).

### 3.6. Mollugin Inhibits Growth of HeLa Cells in a Xenograft Tumor Model

**Figure 6:**
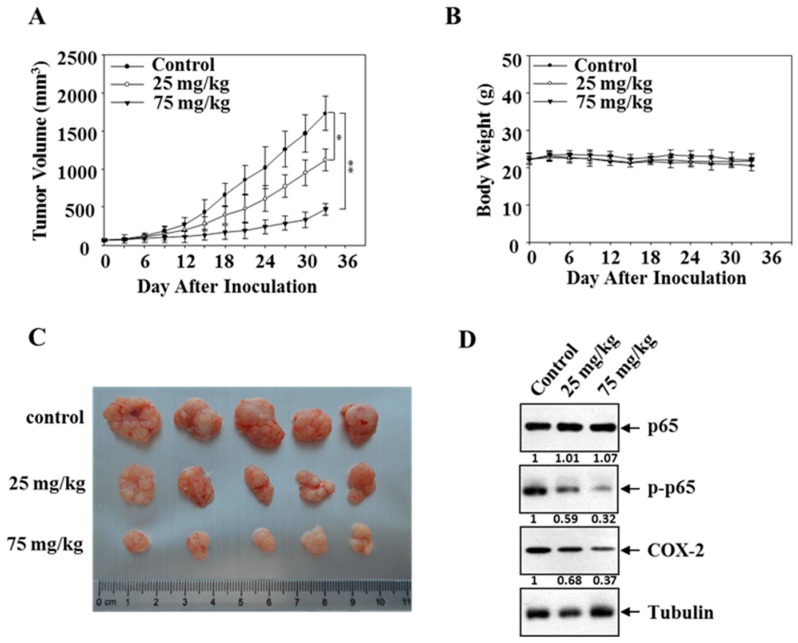
Effect of mollugin on human xenograft model. (**A**,**B**) HeLa cells were implanted s.c. in the left flanks of nude mice, which were administered by gavage three times a week with vehicle (*n* = 5), mollugin (25 mg/kg, *n* = 5) or mollugin (75 mg/kg, *n* = 5) starting from day ten. Tumor volume and mouse weight were measured using calipers and a disital balance, respectively, every three days; (**C**) representative tumor masses of three groups, which were harvested 4 h after the last treatment (and the isolated tumors were photographed); (**D**) western blot analysis of p-p65 and COX-2 in tumor blocks is shown. Tubulin was used as a loading control.

The inhibitory effect of mollugin on the expression of NF-κB target genes and cell proliferation led us to ask whether mollugin affects the growth of HeLa cells in vivo. HeLa cells were subcutaneously implanted in athymic nude mice, and then the mice were treated with mollugin (25 and 75 mg/kg) three times a week until the end of the study. As shown in Figure 6A, mollugin suppressed tumor growth, whereas the body weight did not change (Figure 6B). Tumors were harvested 4 h after the last treatment, and representative tumor masses are shown in Figure 6C. Consistent with the findings in the cultured cells, mollugin significantly reduced the protein expression of p-p65 and COX-2 in the tumors, whereas no significant differences were observed in the tubulin levels (Figure 6D).

## 4. Discussion

*Rubia cordifolia* L., also known as Madder or Indian Madder, is commonly used in Korea as traditional herbal medicine to treat coughs, joint inflammation, and uteritis [20]. Additionally, this plant is used to treat arthritis, dysmenorrhea, and hemostasis in traditional Chinese medicine [21]. Mollugin is a type of naphthohydroquinone from the root of *Rubia cordifolia* L. It has been reported that mollugin can induce tumor cell apoptosis and autophagy, and inhibit cell growth in human oral cancer cells [22,23]. Despite its various pharmacological activities, the molecular mechanism of its anti-tumor effect in HeLa cells has not been adequately elucidated. In this study, we identified mollugin as a potent inhibitor of TNF-α-induced NF-κB activation and investigated how it inhibits NF-κB activation in HeLa cells.

NF-κB dimers are normally maintained in the cytoplasm by interactions with the specific inhibitors, IκBs. After exposure to various agonists that activate the IKK complex, IκBs undergo phosphorylation and rapid ubiquitin-dependent degradation [24]. This releases NF-κB, allowing it to translocate freely into the nucleus [8]. We found that mollugin effectively suppressed TNF-α-induced phosphorylation and nuclear translocation of p65, phosphorylation and degradation of IκBα, and IKK phosphorylation. It has been reported that the anti-apoptotic activity of NF-κB involves the inhibition of TNF-α-induced apoptosis through induction of a variety of anti-apoptotic proteins [22,25]. In the current study, we found that mollugin inhibited the TNF-α-induced expression of anti-apoptotic proteins such as cIAP-1, Bcl-2, and survivin, which are regulated by NF-κB, and their overexpression in many tumors has been associated with tumor cell survival, chemoresistance, and radioresistance. Furthermore, a flow cytometry assay showed that mollugin potentiated TNF-α-induced apoptosis. Caspases are cysteine protease family members that play an important role in apoptosis [26,27,28]. Activated caspases can initiate protein degradation and cell apoptosis irreversibly by cleaving substrate proteins such PARP. We investigated whether mollugin affects TNF-α-induced activation of caspase-3 and PARP.

Indeed, mollugin significantly affected the TNF-α-induced caspase-3 activation and potentiated the TNF-α-induced PARP cleavage. These findings suggest that mollugin potentiates TNF-α-induced caspase activity and cancer cell death, at least in part, via NF-κB inhibition.

NF-κB controls the expression of genes that are involved in cell proliferation [13]. We showed that mollugin suppressed the expression of COX-2, cyclin D1, and c-Myc, which are major mediators of cell proliferation and survival. Furthermore, we performed the EdU incorporation assay and clonogenic assay, to examine whether mollugin affects cell proliferation. Comparable results were obtained by the EdU incorporation assay and clonogenic assay; mollugin significantly decreased the percentage of EdU-positive cells and inhibited cell growth. NF-κB also controls the expression of genes involved in tumor cell invasion and angiogenesis [13,25]. MMP-9 plays a crucial role in tumor invasion and angiogenesis by mediating degradation of the extracellular matrix [29]. Adhesion molecules such as ICAM-1 are regulated by NF-κB and are essential for the adhesion of tumor cells to endothelial cells, and thus mediate tumor cell metastasis [30]. Our results showed that mollugin suppressed not only the expression of MMP-9 and ICAM-1, but also the expression of VEGF, which are major mediators of tumor cell angiogenesis.

**Figure 7:**
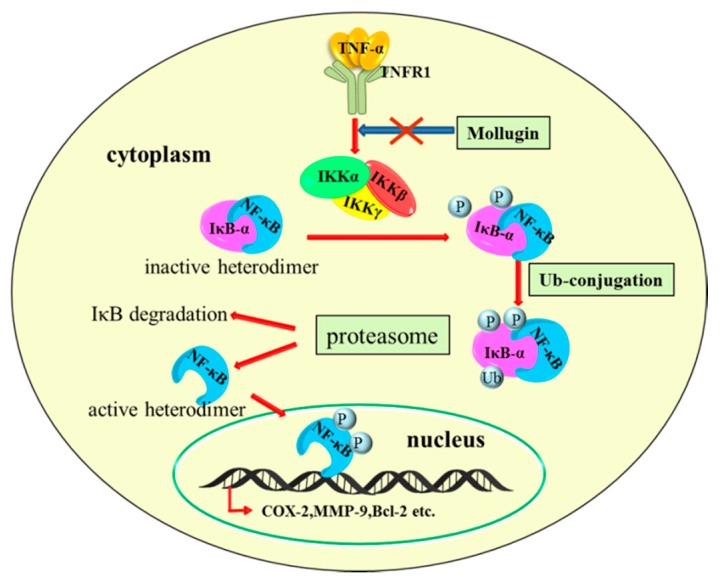
The proposed schematic diagram of signaling pathways for mollugin-mediated, anti-cancer effects. Mollugin blocks TNF-α-induced phosphorylation of the IKK complex as marked by the X symbol in the figure, thereby inhibits the phosphorylation (marked by “P”), ubiquitination (marked by “Ub”), and degradation of IκB-α. This process inhibits the nuclear translocation of NF-κB and downstream gene transcription.

As mollugin suppressed the expression of NF-κB target genes that are associated with cell proliferation, survival, tumor invasion, and angiogenesis, we next examined whether mollugin had anti-tumor activity in vivo, using a xenograft model. HeLa cells were subcutaneously implanted in nude mice without a thymus, and the mice were treated with mollugin (25 or 75 mg/kg). Compared with the vehicle-treated control group, mollugin administration significantly reduced HeLa tumor growth. Furthermore, mollugin suppressed the expression of p-p65 and COX-2 in tumor tissues. Thus, we concluded that mollugin possessed a potent inhibition effect on tumor growth by regulating the NF-κB signaling pathway in the xenograft model. The molecular mechanisms behind the inhibition of NF-κB signaling pathway may include inhibiting the phosphorylation and degradation of IκB-α by inhibiting the phosphorylation of the IKK complex (Figure 7). Taken together, our results suggest that mollugin suppresses NF-κB and NF-κB-regulated gene products, which are associated with proliferative, anti-apoptotic, invasive, and angiogenic effects. Mollugin also inhibited the growth of HeLa cells in a xenograft tumor model. These findings suggest that mollugin may be considered for use as a potential drug for the treatment of human cancer.

## Abbreviations

NF-κB	Nuclear factor-κB
TNF-α	Tumor necrosis factor-α
IκBα	Inhibitor of NF-κB alpha
cIAP-1	Cellular inhibitor of apoptosis protein-1
COX-2	Cyclooxygenase-2
MMP-9	Matrix metalloproteinase-9
ICAM-1	Intercellular cell adhesion molecule-1
VEGF	Vascular endothelial growth factor
